# Nmix: a hybrid deep learning model for precise prediction of 2’-O-methylation sites based on multi-feature fusion and ensemble learning

**DOI:** 10.1093/bib/bbae601

**Published:** 2024-11-16

**Authors:** Yu-Qing Geng, Fei-Liao Lai, Hao Luo, Feng Gao

**Affiliations:** Department of Physics, School of Science, Tianjin University, No. 92 Weijin Road, Nankai District, Tianjin 300072, China; Department of Physics, School of Science, Tianjin University, No. 92 Weijin Road, Nankai District, Tianjin 300072, China; Department of Physics, School of Science, Tianjin University, No. 92 Weijin Road, Nankai District, Tianjin 300072, China; Department of Physics, School of Science, Tianjin University, No. 92 Weijin Road, Nankai District, Tianjin 300072, China; Frontiers Science Center for Synthetic Biology and Key Laboratory of Systems Bioengineering (Ministry of Education), Tianjin University, No. 92 Weijin Road, Nankai District, Tianjin 300072, China; SynBio Research Platform, Collaborative Innovation Center of Chemical Science and Engineering (Tianjin), No. 92 Weijin Road, Nankai District, Tianjin 300072, China

**Keywords:** 2’-O-methylation, multi-feature fusion, deep learning, asymmetric loss, ensemble learning

## Abstract

RNA 2’-O-methylation (Nm) is a crucial post-transcriptional modification with significant biological implications. However, experimental identification of Nm sites is challenging and resource-intensive. While multiple computational tools have been developed to identify Nm sites, their predictive performance, particularly in terms of precision and generalization capability, remains deficient. We introduced Nmix, an advanced computational tool for precise prediction of Nm sites in human RNA. We constructed the largest, low-redundancy dataset of experimentally verified Nm sites and employed an innovative multi-feature fusion approach, combining one-hot, Z-curve and RNA secondary structure encoding. Nmix utilizes a meticulously designed hybrid deep learning architecture, integrating 1D/2D convolutional neural networks, self-attention mechanism and residual connection. We implemented asymmetric loss function and Bayesian optimization-based ensemble learning, substantially improving predictive performance on imbalanced datasets. Rigorous testing on two benchmark datasets revealed that Nmix significantly outperforms existing state-of-the-art methods across various metrics, particularly in precision, with average improvements of 33.1% and 60.0%, and Matthews correlation coefficient, with average improvements of 24.7% and 51.1%. Notably, Nmix demonstrated exceptional cross-species generalization capability, accurately predicting 93.8% of experimentally verified Nm sites in rat RNA. We also developed a user-friendly web server (https://tubic.org/Nm) and provided standalone prediction scripts to facilitate widespread adoption. We hope that by providing a more accurate and robust tool for Nm site prediction, we can contribute to advancing our understanding of Nm mechanisms and potentially benefit the prediction of other RNA modification sites.

## Introduction

To date, over 170 types of post-transcriptional ribonucleic acid (RNA) modifications have been identified, each playing distinct and crucial roles in various biological processes [1–5]. Among these, 2’-O-methylation (Nm) is one of the most prevalent modifications [6]. This modification is catalyzed by the enzyme 2’-O-methyltransferase, which substitutes the hydrogen on the 2’-hydroxyl group with a methyl group [7] (see Supplementary Fig. S1). This modification can occur in all four canonical nucleotides and some non-canonical nucleotides. Studies have shown that Nm is widely distributed across different types of RNA in human. Nm plays pivotal roles and influences RNA splicing, nucleation, stability, and immunogenicity, thereby regulating numerous physiological processes, such as gene expression, tumor immunity and embryonic development [8]. Furthermore, Nm has been implicated in various human diseases, such as Charcot–Marie–Tooth disease, hepatocellular carcinoma and lung adenocarcinoma [9–12]. Despite its importance, the majority of Nm functions and regulatory mechanisms in human remain poorly understood [13, 14]. Therefore, as a critical first step in exploring the potential roles of Nm, accurate identification of Nm sites is essential.

Several experimental methods have been developed to identify Nm sites in RNA [15–19]. To facilitate related studies, the RMBase database was established, containing information on RNA modification sites derived from high-throughput sequencing data [20]. However, experimental identification of Nm sites requires specialized knowledge and expensive equipment, making it time-consuming and labor-intensive. These constraints limit accessibility and fail to meet all the needs of researchers studying Nm sites [21–23]. Thanks to advances in bioinformatics and artificial intelligence [24], numerous computational tools have been developed to predict Nm sites, effectively complementing experimental methods. Chen *et al.* [25] laid the foundation by constructing the first benchmark dataset from RMBase, containing 147 positive and 147 negative samples, and employing a support vector machine classifier. This dataset became instrumental for subsequent methods [26–28], with iRNA-PseKNC(2methyl) [26] notably employing convolutional neural network (CNN) for automatic feature extraction from RNA sequences, exhibits superior performance. As the field progressed, Deep-2‘-O-Me [29] introduced datasets with varying imbalance ratios to better reflect real-world Nm site distribution, combining word2vec and CNN to achieve an area under the receiver operating characteristic curve (AUROC) between 0.89 and 0.92. A significant advancement came from Zhou’s group, who developed NmSEER [30] and NmSEER V2.0 [22] offering the first online Nm site prediction services. Building on this data source, DeepOMe [31] employed one-hot encoding, CNN and bidirectional long short-term memory, achieving an impressive AUROC of 0.993. By using optimal mixed features and a random forest classifier, NmRF [23] achieves an accuracy (ACC) of 86.8%. BERT2OME [32] applied transformer structures from BERT to Chen *et al.*’s dataset, reaching an AUROC of 0.99. A significant leap forward came with i2OM [33], which introduced a large-scale dataset containing 6091 positive samples, far exceeding previous studies. This method employed a two-step feature selection model and uniquely divided data into four sub-datasets based on nucleotide types, achieving an average ACC of 84.3%. Building upon this substantial dataset, H2Opred [21] incorporated an imbalanced test set and employed stacked 1D CNN and Bi-GRU-Att blocks, further improving prediction performance. The latest advancement, Meta-2OM [34], utilizes the i2OM dataset and combines multiple machine learning algorithms with feature encoding techniques for Nm site prediction.

Despite these advancements, several challenges persist in the field of Nm site prediction. Conventional machine learning approaches often involve complex manual extraction and selection of various features, necessitating extensive domain knowledge and potentially affecting model generalization capability due to inherent subjectivity [35–38]. While numerous deep learning models have been developed to address these limitations [21, 26, 29, 31, 32], they often treat sequences merely as strings of nucleotide bases or descriptors, overlooking critical chemical and biological interactions between nucleotides. Furthermore, with the release of RMBase v3.0 [10] and new experimental data [18, 19], there is a pressing need to construct a large-scale, low-redundancy, comprehensive, and imbalanced dataset to develop models with stronger generalization capabilities and robustness. Most importantly, existing computational tools still struggle with false positives on imbalanced datasets, which often represent the most realistic scenarios. For instance, H2Opred [21], despite outperforming other models on an imbalanced independent test set, only achieved an average precision (PRE) of 50.8%. This underscores a critical challenge in real-world applications: Nm sites are naturally imbalanced and diverse, yet current prediction tools perform poorly on such datasets, severely limiting their practical applicability and necessitating the development of more robust approaches.

**Figure 1:**
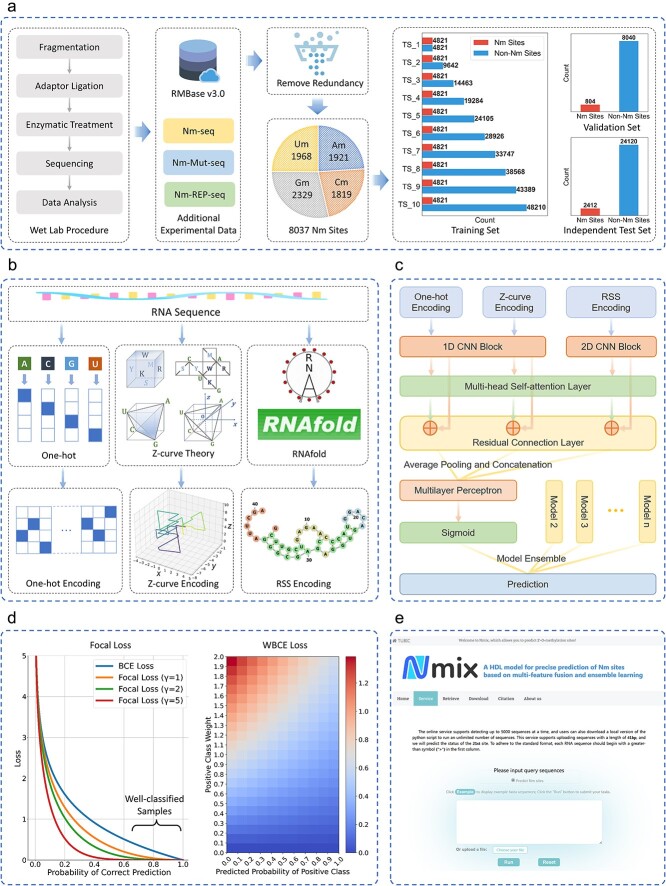
Overview of the present work. (a) The wet lab and bioinformatics pipeline for constructing imbalanced benchmark datasets. (b) Three feature representation and encoding methods for RNA sequence. (c) The model architecture of Nmix. (d) Asymmetric loss function used during training, combining focal loss and WBCE loss. (e) The interface of the web server provided here.

To address these challenges, we presented Nmix, a novel hybrid deep learning model for precise prediction of Nm sites. An overview of the Nmix framework is illustrated in Fig. 1. Nmix incorporated multiple innovations to overcome the limitations of existing methods. First, we constructed a large-scale, low-redundancy dataset from diverse sources, including the latest RMBase v3.0 and recent experimental data. This dataset, coupled with our innovative proposal of 10 training sets with varying positive-to-negative ratios, significantly enhances the model’s generalization and robustness, addressing the common issue of data imbalance in real-world scenarios. Second, Nmix employs a multi-feature fusion approach, uniquely combining one-hot encoding, Z-curve theory and RNA secondary structure (RSS) information to capture both sequence-based and structural characteristics of Nm sites, offering a more comprehensive representation of the underlying biology. Third, we designed a sophisticated neural network architecture that integrated 1D/2D CNNs, multi-head self-attention mechanism and residual connections to effectively extract and process these multi-dimensional features. To tackle the challenge of imbalanced datasets, we implemented an asymmetric loss function and employed Bayesian optimization-based ensemble learning, significantly improving prediction performance, particularly in reducing false positives. Finally, we developed a user-friendly web server to facilitate easy access to Nmix for the research community. By integrating these advanced techniques and addressing key limitations of existing methods, Nmix not only achieved a substantial leap forward in prediction precision and robustness on both benchmark datasets but also significantly outperformed other tools in cross-species generalization test. Nmix can thus serve as an invaluable tool for advancing our understanding of Nm sites and their biological significance across various species, with wide-reaching implications for future research and practical applications.

## Materials and methods

### Benchmark dataset

A well-constructed benchmark dataset is crucial for developing and evaluating computational models in bioinformatics. In this study, we presented a comprehensive workflow for creating a high-quality Nm site prediction dataset, as summarized in Fig. 1a. We aggregated experimentally verified Nm sites in human RNA from multiple sources, including RMBase v3.0 [10], Nm-seq [17] (GSE90164), Nm-Mut-seq [18] (GSE174518), and Nm-REP-seq [19] (GSE157930, GSE198748). This compilation yielded 15 141 Nm sites, representing, to our knowledge, the most extensive collection of experimentally validated Nm sites to date. These sites are distributed across nearly all RNA types and mapped to all 23 pairs of human chromosomes as well as the mitochondrial chromosome. Consistent with previous studies [23, 27, 33], we extracted 41-nucleotide sequences centered on each Nm site (20 nucleotides upstream and downstream) to form positive samples. To ensure sufficient negative samples for constructing imbalanced datasets after redundancy removal, we randomly selected 15 sequences of 41 nucleotides in the vicinity of each Nm site as negative samples. Both positive and negative samples were subjected to redundancy removal using CD-HIT with a similarity threshold of 40%, which is more stringent than the commonly used 80% threshold in previous datasets [21, 23, 25, 33].

The final benchmark dataset contains 8037 positive and 80 370 negative samples, divided into 60% training, 10% validation, and 30% independent testing sets. Departing from conventional balanced training sets, we created ten training subsets (TS_1 to TS_10) with varying positive-to-negative ratios (1:1 to 1:10) to train models under different imbalance levels. We introduced a unified validation set to provide a consistent benchmark for comparing the performance of models trained on these different subsets, thereby assessing model robustness across various imbalance conditions. The independent test set was kept entirely separate to evaluate the final model performance and to ensure fair comparison with other tools. Both validation and independent test sets maintain a 1:10 positive-to-negative ratio, following Pham *et al.*’s approach [21]. We further stratified the dataset into four subsets based on Nm types: 2’-O-methyladenosine (Am), 2’-O-methylcytidine (Cm), 2’-O-methylguanosine (Gm), and 2’-O-methyluridine (Um). Detailed statistics are available in Supplementary Table S1.

### Feature encoding

Feature encoding transforms raw data into a model-compatible format. Our method employed three encoding techniques for RNA sequence representation: one-hot, Z-curve and RSS encodings, as illustrated in Fig. 1b.

#### One-hot encoding

One-hot encoding is a prevalent technique in deep learning for sequence representation. For RNA sequences, it transforms each nucleotide into a unique binary vector: adenine (A) as [1,0,0,0], cytosine (C) as [0,1,0,0], guanine (G) as [0,0,1,0] and uracil (U) as [0,0,0,1].

#### Z-curve encoding

The Z-curve theory is a geometric representation method in bioinformatics for DNA or RNA sequences [39–41]. Based on the symmetry of a regular tetrahedron, it maps any DNA or RNA sequence to a three-dimensional space curve, known as the Z-curve. In this representation, each nucleotide in the RNA sequence corresponds to a point on the Z-curve, with coordinates calculated using the following formulas:


(1) \begin{equation*} {\displaystyle \begin{array}{c}\left\{\begin{array}{c}{x}_n=\left({A}_n+{G}_n\right)-\left({C}_n+{U}_n\right)\\{}{y}_n=\left({A}_n+{C}_n\right)-\left({G}_n+{U}_n\right)\\{}{z}_n=\left({A}_n+{U}_n\right)-\left({G}_n+{C}_n\right)\end{array}\right.\\{}{x}_n,{y}_n,{z}_n\in \left[-N,N\right],n=0,1,2,\dots, N\end{array}} \end{equation*}

The position of the *n*-th node is denoted as $\left({x}_n,{y}_n,{z}_n\right)$, where $N$ represents the length of the sequence studied. ${A}_n$, ${C}_n$, ${G}_n$, and ${U}_n$ represent the cumulative counts of the four nucleobases adenine, cytosine, guanine, and uracil, respectively, in the subsequence from $1$ to $n$ of this RNA sequence. The three components of the Z-curve represent the differences in the four nucleobases across three chemical structures [42]. Specifically, ${x}_n$ represents the distribution of purine/pyrimidine bases along the sequence, ${y}_n$ represents the distribution of amino/keto bases along the sequence, and ${z}_n$ represents the distribution of strong/weak hydrogen bond bases along the sequence (refer to Supplementary Table S2 for details).

#### RNA secondary structure encoding

RSS refers to the base pairings formed when an RNA molecule folds upon itself. This structure is crucial for understanding RNA’s biological functions and properties [43]. Assi *et al.* [6] found that Nm can alter the biological activity of Nm-modified RNAs by modulating their RSS. This suggests that RSS may differ between RNA sequences with and without Nm sites, making it a valuable feature for distinguishing Nm sites. We utilized ViennaRNA Package 2.0 [44], a popular tool for RSS computation, to generate structural expressions for each sequence. In these expressions, matching ‘(’ and ‘)’ denote base pairs. We encoded RSS as a square matrix, where matrix dimensions equal the RNA sequence length. Matrix elements (*x*, *y*) and (*y*, *x*) are set to 1 if the *x*-th and *y*-th bases pair, and 0 otherwise.

### Model architecture of Nmix

In this study, we adopted a hybrid neural network architecture comprising several key components, as shown in Fig. 1c. The model includes CNN blocks, a multi-head self-attention layer, residual connections and a multilayer perceptron (MLP). To enhance the overall predictive performance, we employed model ensemble techniques, combining predictions from multiple models trained through cross-validation.

#### Convolutional neural network blocks

Our model incorporates both 1D and 2D CNN blocks to process different encodings of RNA sequences. For one-hot and Z-curve encodings, we implemented two-layer 1D CNN blocks. These blocks are designed to capture local and more expansive patterns in the sequence data. For the RSS encoding, we employed a two-layer 2D CNN block, which extracts hierarchical spatial features from the RSS representation [45–47]. In both CNN block types, we utilized batch normalization after each convolutional layer to stabilize the learning process and accelerate training. We also employed the parametric rectified linear unit (PReLU) activation function, which adapts its parameters during training, allowing the model to learn more complex and nuanced representations of the data [48]. For detailed information about the CNN blocks, please refer to the supplementary information.

#### Multi-head self-attention layer

The output of the CNN blocks is then fed into a multi-head self-attention layer. The self-attention mechanism allows the model to attend to different parts of the sequence simultaneously [49]. In our model, we utilized four heads, each with its own set of learned linear transformations to generate query, key and value vectors. The formula is as follows:


(2) \begin{equation*} {\displaystyle \begin{array}{c}\left\{\begin{array}{c}{Q}_i=X{W}_i^Q\\{}{K}_i=X{W}_i^K\\{}{V}_i=X{W}_i^V\end{array}\right.\end{array}} \end{equation*}

where $X$ is the input matrix, and ${W}_i^Q$, ${W}_i^K$ ​and ${W}_i^V$ are parameter matrices for the *i*-th head.

The scaled dot-product attention function is mathematically presented as:


(3) \begin{equation*} {\displaystyle \begin{array}{c}\mathrm{Attention}\left(Q,K,V\right)=\mathrm{softmax}\left(\frac{Q{K}^T}{\sqrt{d_k}}\right)V\end{array}} \end{equation*}

This function scales the dot-product of $Q$ and $K$ by $\frac{1}{\sqrt{d_k}}$ to prevent excessively large values during the computation, where ${d}_k$ is the dimension of the key vectors, before applying a softmax function to obtain the weights on the values. The output for each head, denoted as ${head}_i$, is computed as:


(4) \begin{equation*} {\displaystyle \begin{array}{c}\ {\mathrm{head}}_i=\mathrm{Attention}\left({Q}_i,{K}_i,{V}_i\right)\end{array}} \end{equation*}

The outputs of four heads are then concatenated and subsequently transformed by the weight matrix ${W}^O$, as shown by the equation:


(5) \begin{equation*} {\displaystyle \begin{array}{c}\mathrm{MultiHead}(X)=\mathrm{Concat}\ \left(\ \!{\mathrm{head}}_1,{\mathrm{head}}_2,{\mathrm{head}}_3,{\mathrm{head}}_4\right){W}^O\end{array}} \end{equation*}

By projecting the input vectors into different subspaces (heads), the model can focus on different positions, capturing various features and dependencies in the RNA sequence.

#### Residual connection layer

With the increasing depth of neural networks, issues such as vanishing gradients and exploding gradients make the training of deep networks challenging [50, 51]. To address these problems, residual connections have been introduced in the model. The formula is as follows:


(6) \begin{equation*} {\displaystyle \begin{array}{c}Y=X+\mathrm{MultiHead}(X)\end{array}} \end{equation*}

where $X$ represents the output of the CNN block and is also the input of the multi-head attention layer.

#### Multilayer perceptron layer

The MLP layer serves as the final processing stage of our model, transforming the extracted features into a format suitable for classification [48]. It comprises seven hidden layers with progressively decreasing output sizes from 128 to 2. Dropout (with a rate of 0.05) and PReLU activation functions are incorporated between layers for regularization and nonlinearity. The final output is mapped to a probability score using the sigmoid function, indicating the likelihood of the center base being an Nm site.

#### Ensemble learning

Ensemble learning is a methodology that combines multiple baseline models to create a more powerful composite model. This approach not only enhances generalization capability and robustness but also reduces the risk of overfitting due to the diversity of the constituent models [52–54]. Our five-fold cross-validation approach (see Training configuration section) yielded five distinct models, each trained on different subsets of the data. Common ensemble methods include hard voting (majority rule) and soft voting (averaging probability predictions). However, these methods can be biased toward weaker models and may not optimally combine the strengths of individual learners.

To address these limitations, we employed Bayesian optimization to determine the optimal weights for our ensemble, thereby enhancing the contribution of each model [55]. Bayesian optimization is a sequential model-based technique, ideal for optimizing complex objectives, such as maximizing the Matthews correlation coefficient (MCC) on our validation set by finding optimal model weights. The ensemble prediction is given by:


(7) \begin{equation*} {\displaystyle \begin{array}{c}\hat{y}={w}_1{f}_1(x)+{w}_2{f}_2(x)+{w}_3{f}_3(x)+{w}_4{f}_4(x)+{w}_5{f}_5(x)\end{array}} \end{equation*}

where $\left\{{w}_1,{w}_2,{w}_3,{w}_4,{w}_5\right\}$ are the model weights, constrained by ${\sum}_{i=1}^5\kern0.1em {w}_i=1$ and ${w}_i\ge 0$. The goal is to maximize MCC:


(8) \begin{equation*} {\displaystyle \begin{array}{c}\left\{{w}_1^{\ast },{w}_2^{\ast },{w}_3^{\ast },{w}_4^{\ast },{w}_5^{\ast}\right\}=\underset{\left\{{w}_1,{w}_2,{w}_3,{w}_4,{w}_5\right\}}{\arg \mathit{\max}}\mathrm{MCC}\left(\hat{y},{y}_{\mathrm{true}}\right)\end{array}} \end{equation*}

Bayesian optimization uses a Gaussian process as a surrogate model to approximate the relationship between the weights and MCC. The optimization iteratively selects candidate weights by maximizing the expected improvement acquisition function, which balances exploration and exploitation. This process efficiently identifies the optimal weights, leading to a more robust and accurate ensemble by optimally combining the strengths of each model.

### Training methodology

#### Asymmetric loss function

To modulate the model’s focus on different samples during training, we utilized an asymmetric loss function that adjusts the relative importance of positive and negative samples through a weighted binary cross-entropy (WBCE) and focuses on hard-to-classify examples via a focal loss component [56, 57]. The asymmetric loss function is defined as:


(9) \begin{equation*} {\displaystyle \begin{array}{c}\mathcal{L}=\frac{1}{N}\sum_{i=1}^N\kern0.1em {\left(1-{p}_{t,i}\right)}^{\gamma}\cdotp{\mathcal{L}}_{\mathrm{WBCE}}\left({p}_i,{y}_i\right)\end{array}} \end{equation*}

where ${p}_i=\sigma \left({x}_i\right)$ is the predicted probability obtained via the sigmoid function, ${y}_i\in \{0,1\}$ is the ground truth label, and ${p}_{t,i}={p}_i{y}_i+\left(1-{p}_i\right)\left(1-{y}_i\right)$ represents the model’s confidence in the correct class for the $i$-th sample in a batch of $N$ samples. The focal loss component is characterized by the factor ${\left(1-{p}_{t,i}\right)}^{\gamma }$, where $\gamma \ge 0$ is a focusing parameter that modulates the importance of well-classified versus misclassified examples, as shown in the left panel of Fig. 1d. We set $\gamma =2$ during training, a commonly used value that effectively balances the focus on hard examples. The WBCE loss for a single instance is given by:


(10) \begin{equation*} {\displaystyle \begin{array}{c}\begin{array}{c}{\mathcal{L}}_{\mathrm{WBCE}}\left({p}_i,{y}_i\right)=-\left[w\cdotp{y}_i\cdotp \log \left({p}_i\right)+\left(1-{y}_i\right)\cdotp \log \left(1-{p}_i\right)\right]\end{array}\end{array}} \end{equation*}

where $w$ is a weighting factor that adjusts the importance of the positive class. As illustrated in the right panel of Fig. 1d, when the true label ${y}_i=1$, the WBCE loss increases with higher positive class weights $w$, particularly when the predicted probability ${p}_i$ is low.

#### Training configuration

We implemented our model using PyTorch and conducted training using a five-fold cross-validation approach. For each fold, we employed an early stopping mechanism based on the corresponding validation set. Training was terminated if the model’s MCC showed no improvement for 10 consecutive epochs on this validation set. This approach helps prevent overfitting and reduces unnecessary computation time. The AdamW optimizer was employed with an initial learning rate of 0.001 and weight decay of ${10}^{-6}$. This optimizer combines the benefits of Adam’s adaptive learning rates with weight decay regularization, helping to mitigate overfitting while maintaining efficient training dynamics [58]. We coupled this with a cosine annealing learning rate scheduler, which cyclically varies the learning rate, allowing the model to escape local minima and potentially converge to better optima [59]. Additional training parameters included a maximum gradient norm of 10 to prevent gradient explosions and a batch size of 64. All experiments were conducted on a server equipped with four NVIDIA GeForce RTX 3090 GPUs and an Intel(R) Xeon(R) Platinum 8160 CPU.

### Performance evaluation strategies

In this study, we employed a comprehensive set of evaluation metrics widely used in machine learning and bioinformatics research [60, 61], including ACC, recall, PRE, MCC, AUROC, and F1-score, as calculated in Formula (11). Detailed explanations of these metrics are provided in the supplementary information.


(11) \begin{equation*} {\displaystyle \begin{array}{c}\left\{\begin{array}{c} ACC=\frac{TP+ TN}{TP+ TN+ FP+ FN}\\{} Recall=\frac{TP}{TP+ FN}\\{} PRE=\frac{TP}{TP+ FP}\\{} MCC=\frac{TP\times TN- FP\times FN}{\sqrt{\left( TP+ FP\right)\left( TP+ FN\right)\left( TN+ FP\right)\left( TN+ FN\right)}}\\{} AUROC=\int TPRd(FPR)\\{}F1- score=2\times \frac{PRE\times Recall}{PRE+ Recall}\end{array}\right.\end{array}} \end{equation*}

## Results

### Evaluating Nmix performance across training set compositions and positive class weights

**Figure 2:**
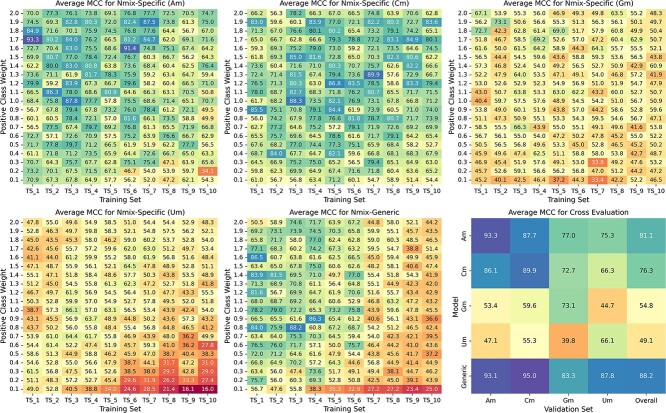
MCC for different combinations of training set compositions and positive class weights, and cross-evaluation.

Nmix performance is significantly influenced by two key parameters: the positive-to-negative ratio in training sets and the positive class weight in the loss function. To comprehensively explore their effects, we conducted an extensive analysis using 200 parameter combinations derived from ten training sets (TS_1 to TS_10) with varying positive-to-negative ratios, each paired with 20 positive class weights ranging from 0.1 to 2.0 in 0.1 increments. This weight range was chosen to explore both under- and over-emphasis of positive samples in imbalanced datasets. We applied these combinations to train two types of models: Nmix-specific and Nmix-generic. Nmix-specific models were trained on individual nucleotide subsets (Am, Cm, Gm, and Um), each focusing on a specific type of Nm. In contrast, the Nmix-generic model was trained on the overall dataset, encompassing all nucleotide types. For each parameter combination, we employed five-fold cross-validation and evaluated all models on our benchmark dataset’s unified validation set. We chose MCC as the key performance metric due to its balanced assessment capability for imbalanced datasets. Figure 2 presents the comprehensive results, including a cross-evaluation using the parameter combinations that yielded the highest average MCC for each model type.

Nmix-specific models showed significant performance variations across nucleotide types, with Am and Cm models outperforming Gm and Um models. Cross-evaluation revealed that the Nmix-generic model achieved the highest MCC on Cm, Gm and Um subsets. This suggests that performance disparities are primarily attributable to dataset differences rather than inherent biological distinctions. In essence, this observation indicates that Nm modifications are not directly associated with specific nucleotide bases, consistent with Nm occurring on the ribose sugar rather than on specific nucleotide sites. These results align with Pham *et al.*’s observations [21], reinforcing our understanding that Nm does not have a strong correlation with nucleotide identity.

Most models achieved superior performance with moderately imbalanced training sets. Nmix-generic and Nmix-specific (Cm, Gm, Um) models reached peak performance at positive-to-negative ratios of 1:3, 1:7, 1:2, and 1:3, respectively, with MCC values of 88.2%, 89.9%, 73.1% and 66.1%. However, extremely imbalanced training sets (1:8 to 1:10) led to significant performance degradation. Analyzing the impact of positive class weights, we observed no consistent trend in MCC scores. Interestingly, for more imbalanced training sets, the optimal positive class weight for peak performance sometimes decreased. For example, Nmix-generic’s optimal weights for 1:1, 1:2 and 1:3 ratios were 1.6, 1.4 and 0.8, respectively. This suggests that our model can effectively learn significant differences between positive and negative samples, even at lower positive class weights, while also indicating some uncertainty in handling class imbalance.

### Ablation study

To rigorously assess the contribution of each component in the Nmix model, we conducted a comprehensive ablation study based on both the validation set and the independent test set, focusing on two main aspects: feature ablation and model architecture ablation. The feature ablation study systematically evaluated different combinations of three feature encoding methods: one-hot, Z-curve and RSS. The model architecture ablation created multiple variants by systematically removing key components of the Nmix architecture to isolate the contribution of each architectural element. Results for Nmix-generic are in Tables 1 and 2, with Nmix-specific results in Supplementary Tables S3 and S4.

**Table 1:** Results of feature ablation study for Nmix-generic

Dataset	Model	ACC (%)	Recall (%)	PRE (%)	AUROC (%)	MCC (%)	F1-score (%)
Validation set	One-hot	90.5	58.2	48.8	87.5	47.9	52.8
	Z-curve	92.0	49.2	63.4	89.3	49.2	50.5
	RSS	90.4	1.2	7.9	57.9	0.8	1.7
	One-hot + Z-curve	90.8	59.4	49.8	87.0	49.3	54.1
	One-hot + RSS	93.4	70.3	63.1	91.2	63.0	66.5
	Z-curve + RSS	91.5	53.2	60.6	88.9	47.0	47.5
	One-hot + Z-curve + RSS (Nmix)	**98.0**	**91.5**	**87.3**	**99.2**	**88.2**	**89.3**
Independent test set	One-hot	90.7	58.4	49.7	87.3	48.7	53.4
	Z-curve	92.3	50.6	65.2	89.1	50.9	52.1
	RSS	90.4	0.9	9.4	57.7	1.0	1.4
	One-hot + Z-curve	91.1	59.4	51.2	87.0	50.2	54.9
	One-hot + RSS	93.8	72.3	64.4	91.3	64.8	68.1
	Z-curve + RSS	91.3	52.7	58.7	89.1	45.4	46.1
	One-hot + Z-curve + RSS (Nmix)	**98.5**	**95.6**	**89.0**	**99.6**	**91.4**	**92.1**

Note: the highest score in each column is shown in bold.

**Table 2:** Results of model architecture ablation study for Nmix-generic

Dataset	Model	ACC (%)	Recall (%)	PRE (%)	AUROC (%)	MCC (%)	F1-score (%)
Validation set	Nmix-NoCNN	88.8	16.8	30.0	72.2	16.3	20.5
	Nmix-SingleCNN	88.5	46.1	39.1	81.5	36.1	42.1
	Nmix-NoAttention	88.6	54.0	41.2	83.7	40.8	46.3
	Nmix-NoResidual	90.6	50.7	47.9	85.9	44.0	49.0
	Nmix-MLP3	91.0	53.6	52.4	84.5	47.7	52.2
	Nmix-MLP5	93.1	64.0	60.2	89.8	58.1	61.6
	Nmix-NoFocal	95.2	77.3	72.7	94.7	72.3	74.8
	Nmix-full	**98.0**	**91.5**	**87.3**	**99.2**	**88.2**	**89.3**
Independent test set	Nmix-NoCNN	88.9	16.6	29.9	71.9	16.3	20.4
	Nmix-SingleCNN	88.4	45.9	38.8	81.1	35.7	41.8
	Nmix-NoAttention	88.7	53.9	41.5	83.7	41.0	46.5
	Nmix-NoResidual	90.6	49.6	48.0	85.7	43.5	48.5
	Nmix-MLP3	91.1	53.9	52.6	84.3	48.1	52.5
	Nmix-MLP5	93.5	64.8	62.0	90.0	59.6	63.0
	Nmix-NoFocal	95.5	78.9	73.8	94.8	73.8	76.2
	Nmix-full	**98.5**	**95.6**	**89.0**	**99.6**	**91.4**	**92.1**

Note: the highest score in each column is shown in bold.

Feature ablation analysis reveals that the Nmix model, integrating all three feature encoding methods, significantly outperforms single-feature or dual-feature combinations across all metrics, underscoring the importance of feature fusion. While RSS features alone perform poorly, their combination with one-hot features substantially improves performance, increasing MCC by 16.1% on the independent test set compared to one-hot alone. This indicates that RSS features provide unique and valuable information. However, the combination of Z-curve and RSS features did not enhance performance as expected, suggesting that feature interactions are complex and not simply additive. Architecturally, removing any component degrades performance. Models lacking CNN layers (Nmix-NoCNN) or using only a single CNN layer (Nmix-SingleCNN) perform worst, with MCC of 16.3% and 35.7%, respectively, on the independent test set, highlighting the crucial role of multi-layer CNNs in feature extraction. Removing the self-attention mechanism or residual connection also significantly reduces performance, validating their importance in capturing long-range dependencies and facilitating gradient flow. Regarding the depth of MLP layers, the results indicate that appropriately increasing the depth (from Nmix-MLP3 to Nmix-MLP5 to Nmix-Full) can improve model performance. Although Nmix-NoFocal performs well, the complete Nmix model achieves substantially better results, indicating that the focal loss function enhances the model’s ability to learn from difficult-to-classify samples.

Notably, Nmix shows similar results on validation and independent test sets, indicating good generalization without overfitting. These findings highlight each component’s importance in Nmix and demonstrate the effectiveness of multi-feature fusion and sophisticated architectures in RNA modification prediction.

### Optimizing model performance through ensemble methods

**Figure 3:**
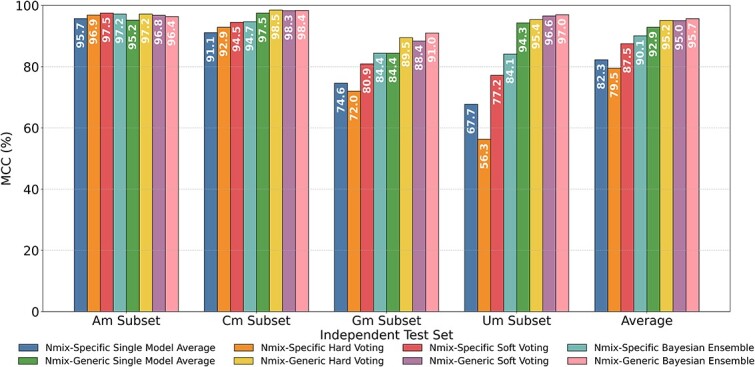
Comparison of different model ensemble methods with the baseline.

To further enhance our models’ predictive precision and generalization capability, we employed ensemble methods. As previously described, we applied five-fold cross-validation to train both Nmix-specific and Nmix-generic models, generating five distinct sub-models in each training iteration. We then implemented three different model ensemble strategies on these sub-models: hard voting, soft voting and Bayesian optimization-based ensemble learning. For baseline comparison, we also calculated the average of individual prediction results from the five sub-models. Consistent with the previous analysis, we selected the MCC as the key performance metric, given its ability to provide a more comprehensive and reliable measure on imbalanced datasets. Figure 3 visually illustrates the MCC performance of these methods across four nucleotide subsets (Am, Cm, Gm, Um), along with their average MCC values.

The effectiveness of model ensemble methods varies with baseline model performance. For models with excellent baseline performance (MCC > 95%), such as Nmix-specific and Nmix-generic on the Am subset and Nmix-generic on the Cm subset, model ensemble provides modest improvements, with soft and hard voting typically performing best due to minimal inter-model disparities. For models with very good baseline performance (80% < MCC < 95%), including Nmix-specific on Cm and Nmix-generic on Gm and Um subsets, model ensemble yields significant gains. In these cases, Bayesian optimization-based ensemble learning demonstrates advantages, improving MCC by 3.6%, 6.6%, and 3.7%, respectively, over baseline. For models with lower baseline MCC, such as Nmix-specific on Gm (74.6%) and Um (67.7%) subsets, Bayesian optimization-based ensemble learning excels, improving MCC by 9.8% and 16.4% over baseline, and outperforming soft voting by 3.5% and 6.9%, respectively.

Analyzing the average performance across all subsets, the Bayesian optimization-based ensemble learning method consistently demonstrates superior results, surpassing baseline performance by 7.8% and 2.8% in Nmix-specific and Nmix-generic models, respectively. Interestingly, hard voting ensemble demonstrates varying effectiveness across model types. While it achieves the second-highest average MCC (95.2%) in Nmix-generic, surpassing soft voting, it underperforms the baseline in Nmix-specific (79.5% versus 82.3%). This disparity highlights hard voting’s potential instability with models of varying performance levels, emphasizing the importance of carefully considering base model characteristics when selecting an ensemble method.

### Comparison with existing methods

#### Performance comparison on benchmark datasets

To validate the superiority of Nmix, we conducted a comprehensive comparison with existing methods. We utilized both our newly proposed benchmark dataset and the dataset introduced by Pham *et al.* [21], which was previously considered the most comprehensive and robust. For both datasets, we trained Nmix on the designated training sets and evaluated its performance on completely separate and independent test sets. This strict separation between training and test data was maintained when comparing Nmix with other models. While we considered all existing methods, earlier works often used significantly smaller datasets and lacked publicly available code or prediction tools, making fair comparisons challenging. Therefore, we focused our comparison on three state-of-the-art (SOTA) tools that used the same training set as Pham *et al.*’s benchmark dataset: i2OM [33], H2Opred [21], and Meta-2OM [34].

For the comparison on Pham *et al.*’s dataset, Table 3 reports the average performance of all methods on the independent test set, based on five models trained through five-fold cross-validation. Notably, Nmix did not use model ensemble methods for this comparison. H2Opred is divided into H2Opred-Specific and H2Opred-Generic, analogous to our Nmix-specific and Nmix-generic, representing models trained on individual nucleotide subsets and the entire training set, respectively. Nmix-generic consistently outperforms Nmix-specific, which is similar to the relationship between H2Opred-Generic and H2Opred-Specific. On average, Nmix-generic surpasses the best existing method (H2Opred-Generic) by 6.2% in ACC, 33.1% in PRE, 4.2% in AUROC, 24.7% in MCC, and 23.3% in F1-score. Across specific nucleotide subsets, Nmix-generic demonstrates remarkable enhancements, particularly in PRE for Um (48.1% increase) and Cm (34.5% increase). Among the other three methods, H2Opred-Specific and H2Opred-Generic generally outperforms Meta-2OM, with both showing considerable improvement over i2OM.

**Table 3:** Performance comparison based on the independent test set proposed by Pham *et al*.

Subset	Method	ACC (%)	Recall (%)	PRE (%)	AUROC (%)	MCC (%)	F1-score (%)
Am	i2OM	46.1	90.8	13.5	86.2	19.2	23.5
	H2Opred-specific	91.4	84.2	51.7	94.6	61.8	64.1
	H2Opred-generic	92.1	86.4	54.2	95.4	64.6	66.6
	Meta-2OM	88.4	67.7	41.7	85.8	47.1	51.5
	Nmix-specific	93.3	85.6	67.5	96.1	71.9	73.6
	Nmix-generic	**97.0**	**92.1**	**80.8**	**98.5**	**84.4**	**85.5**
Cm	i2OM	88.4	77.4	42.5	91.5	51.8	54.9
	H2Opred-specific	90.8	81.3	49.7	93.5	59.0	61.7
	H2Opred-generic	91.8	82.1	53.0	94.9	61.8	64.4
	Meta-2OM	87.8	74.3	40.9	88.9	49.2	52.7
	Nmix-specific	91.6	80.7	58.3	94.3	63.8	66.3
	Nmix-generic	**98.1**	**93.0**	**87.5**	**99.5**	**89.0**	**89.8**
Gm	i2OM	88.5	83.0	43.1	93.4	54.5	56.8
	H2Opred-specific	90.9	84.5	50.0	94.5	60.6	62.8
	H2Opred-generic	91.0	86.2	50.3	95.8	61.6	63.5
	Meta-2OM	85.0	54.7	31.4	79.0	33.6	39.8
	Nmix-specific	89.3	84.6	52.7	93.7	61.0	63.1
	Nmix-generic	**96.1**	**94.9**	**73.3**	**98.9**	**81.3**	**82.3**
Um	i2OM	77.9	59.7	22.7	77.4	26.6	32.9
	H2Opred-specific	85.6	83.5	37.1	91.7	49.4	51.3
	H2Opred-generic	89.5	78.6	45.5	92.8	54.7	57.6
	Meta-2OM	80.6	82.3	29.7	89.0	41.5	43.6
	Nmix-specific	86.9	**90.7**	45.8	95.3	58.4	59.6
	Nmix-generic	**97.9**	82.2	**93.6**	**98.7**	**86.6**	**87.4**
Average	i2OM	75.2	77.7	30.4	87.1	38.0	42.0
	H2Opred-specific	89.6	83.3	47.1	93.5	57.7	59.9
	H2Opred-generic	91.1	83.3	50.7	94.7	60.6	63.0
	Meta-2OM	85.4	69.8	35.9	85.7	42.8	46.9
	Nmix-specific	90.3	85.4	56.1	94.9	63.8	65.7
	Nmix-generic	**97.3**	**90.6**	**83.8**	**98.9**	**85.3**	**86.3**

Note: the highest score in each column is shown in bold.

**Figure 4:**
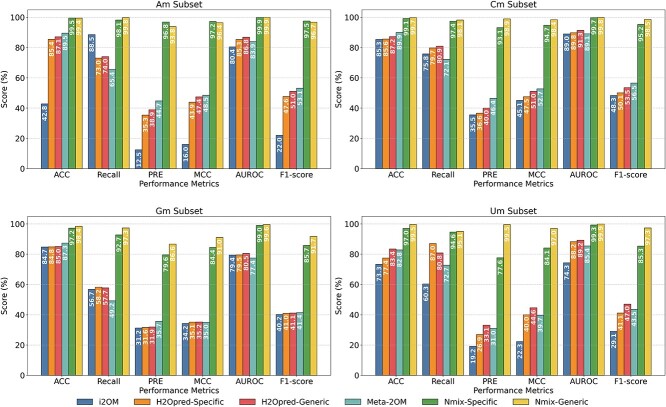
Evaluation metrics on our proposed independent test set.

For our proposed benchmark dataset, we employed available prediction tools from existing methods, which typically represent their best performance. Among the five currently available tools (NmSEER [30], NmSEER V2.0 [22], i2OM [33], H2Opred [21], Meta-2OM [34]), NmSEER and NmSEER V2.0 yielded near-random prediction results, possibly due to their small training sets. Consequently, we compared i2OM, H2Opred and Meta-2OM with Nmix on our independent test set, as shown in Fig. 4. The figure demonstrates that the Nmix-generic model achieves superior performance across all test datasets. Compared to the best metrics from other methods in each test subset, Nmix-generic exhibits substantial improvements, particularly in MCC, PRE and F1-score. In the Am subset, Nmix-generic surpasses the second-best method by 47.9%, 49.1%, and 43.6% in MCC, PRE, and F1-score, respectively. For the Cm subset, Nmix-generic’s MCC improves by 45.7% and PRE by 52.5%. In the Gm subset, MCC and PRE increase by 55.8% and 50.9%, respectively. Other methods perform poorly on the Um subset, where Nmix achieves the most significant advancements: MCC, PRE and F1-score increase by 52.4%, 66.4%, and 50.3%, while ACC, recall and AUROC improve by 16.1%, 8.1%, and 10.7%, respectively. On average, compared to the second-highest values for each metric, Nmix-generic showed improvements of 11.8% in ACC, 23.1% in recall, 60.0% in PRE, 51.1% in MCC, 7.7% in AUROC, and 47.4% in F1-score. Among other methods, H2Opred-Generic generally outperforms H2Opred-Specific and i2OM, while Meta-2OM surpasses or approaches H2Opred-Generic in certain metrics. Nmix-generic consistently outperforms Nmix-specific across all subsets except Am, aligning with our previous observations.

These comprehensive and significant performance enhancements underscore the stability and excellence of the Nmix model. They also demonstrate its robust generalization capability across different nucleotide types of Nm sites and evaluation criteria, establishing Nmix as a reliable and advanced computational tool for accurate identification and prediction of Nm sites. Overall, Nmix’s superior performance across diverse datasets and metrics highlights its potential to significantly advance the field of Nm site prediction, offering researchers a more accurate and versatile tool for RNA modification studies.

#### Exploring cross-species generalization capability

**Figure 5:**
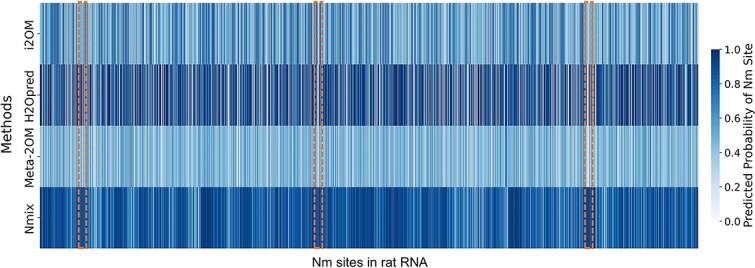
Prediction results of i2OM, H2Opred, meta-2OM, and Nmix for Nm sites in rat RNA.

Given the high conservation of Nm sites across multiple species [13], we sought to evaluate the cross-species generalization ability of Nmix in comparison to i2OM [33], H2Opred [21], and Meta-2OM [34]. This conservation suggested that models trained on human data might potentially predict Nm sites in other species, providing an opportunity to assess their broader applicability and robustness. We obtained 3412 experimentally verified Nm sites in rat (*Rattus norvegicus*) RNA from RMBase v3.0, which contains the second-largest number of Nm sites after human data. We used Nmix and the other three tools to predict these samples. As the dataset included all four types of Nm sites (Am, Cm, Gm, Um), we employed the generic versions of Nmix and H2Opred. The results are illustrated in Fig. 5, where the horizontal axis represents the Nm sites in rat RNA, and the vertical axis shows the four different prediction methods. Each cell in the figure represents the prediction probability (ranging from 0 to 1) of the corresponding method for each Nm site. Notably, in the highlighted rectangular areas, Nmix successfully identified Nm sites that the other three methods failed to predict accurately. The figure clearly demonstrates that Nmix achieved superior predictive performance. H2Opred performed better than i2OM and Meta-2OM but was outperformed by Nmix. Overall, Nmix achieved a recall of 93.8%, significantly surpassing the recall rates of i2OM (48.7%), H2Opred (67.0%), and Meta-2OM (33.8%). This substantial improvement in cross-species prediction recall underscores Nmix’s robust generalization capability, suggesting its potential for accurate Nm site identification across diverse species beyond its training data.

### Visualization with T-distributed stochastic neighbor embedding

**Figure 6:**
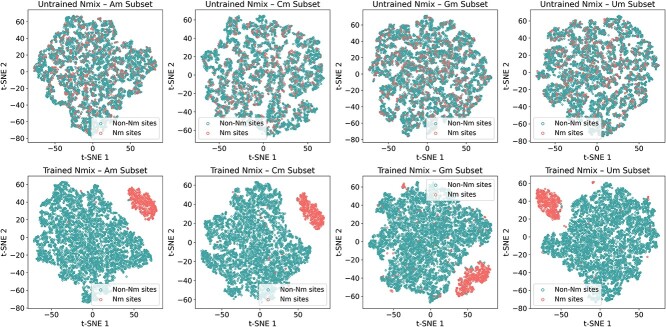
t-SNE visualization results of the features extracted by untrained Nmix and trained Nmix from the independent test set.

T-distributed stochastic neighbor embedding (t-SNE) is a powerful dimensionality reduction technique specifically designed to visualize high-dimensional data in a lower dimensional space, typically two or three dimensions [62, 63]. We applied t-SNE to visualize high-dimensional features extracted just before the MLP in both untrained and trained Nmix models. Figure 6 shows the t-SNE plots for four subsets (Am, Cm, Gm, Um) of the independent test set, with Nm sites represented by red circles and non-Nm sites by green circles. In the untrained model, Nm and non-Nm sites are largely intermixed across all subsets. Conversely, the trained Nmix visualizations reveal distinct clustering of the two site types. This clear separation demonstrates that the Nmix model, after training, effectively distinguishes between Nm and non-Nm sites.

### Web server

To make Nm site prediction tools readily accessible to the scientific community, we have developed and deployed a user-friendly web server for Nmix, freely available at https://tubic.org/Nm. Built on a robust LAMP stack (Linux, Apache, MariaDB, and PHP), the server can process up to 5000 sequences simultaneously, utilizing the optimized Nmix Python script for rapid and accurate predictions. Each submission is assigned a unique task ID, and results are stored on the server for one month, enabling easy tracking and retrieval. For users requiring higher throughput, a downloadable standalone script is available from https://github.com/tubic/Nmix for local execution with unlimited sequence analysis. By offering both online and offline options, the Nmix web server caters to a wide range of research needs, from small-scale exploratory analyses to large-scale genomic studies, aiming to accelerate research in RNA modification. The user interface of the web server is shown in Fig. 1e.

## Conclusion

This study presented Nmix, an advanced computational tool for accurate prediction of Nm sites in human RNA. We first constructed a new imbalanced dataset by integrating experimentally verified Nm sites from various sources, resulting in a collection that is more extensive and diverse than previous datasets while maintaining low redundancy. The RNA sequence samples were then encoded using an innovative multi-feature fusion approach, combining one-hot, Z-curve and RSS encodings. Notably, this is the first application of RSS encoding in Nm site prediction research, allowing us to effectively capture multidimensional features. These encoded features were input into our carefully designed hybrid deep learning architecture, which incorporates sophisticated components such as CNNs, self-attention mechanism and residual connection. This complex model processes the multi-feature inputs to generate the final Nm site predictions. Ablation studies demonstrated that each module contributes significantly to the model’s performance, validating our architectural choices. Furthermore, by exploring various positive-to-negative sample ratios and positive class weights, we optimized the model’s performance across different Nm types, revealing the necessity of these parameter adjustments in handling imbalanced datasets.

Our research revealed that Nmix-specific models showed considerable performance variations across different Nm types, while Nmix-generic performed well across all types. This finding suggests that prediction performance differences primarily stem from dataset characteristics rather than inherent biological differences among Nm types. Nmix exhibited excellent performance on two imbalanced independent test sets that closely resemble real-world conditions, significantly outperforming existing tools, particularly in terms of PRE and MCC. This demonstrates its potential for practical applications. Furthermore, Nmix showed superior cross-species generalization capability, significantly outperforming other tools and corroborating the high conservation of Nm sites across species. These findings provide a reliable tool for large-scale identification of RNA Nm sites, laying a foundation for deeper understanding of RNA modification functions and regulatory mechanisms, with potential applications in RNA-related disease research and drug development. To facilitate accessibility and promote wider adoption in the research community, we developed a web server for Nmix and provided open-source code for local predictions. This user-friendly platform allows researchers to easily predict Nm sites in the RNA sequences of interest, potentially accelerating discoveries in RNA modification research and related fields.

While this study represents a significant advance, it is important to acknowledge certain limitations. The model’s performance lacks clear patterns under different parameter combinations, indicating potential dataset biases and model uncertainties. Due to computational resource constraints, we were unable to explore more imbalanced training sets and a wider range of positive class weight variations, an exploration that could potentially yield valuable insights [64]. Moreover, the complexity of the model makes it challenging to directly interpret its decision-making process, potentially limiting our ability to derive meaningful biological insights. This ‘black box’ nature is a common challenge in deep learning models [65]. Nevertheless, Nmix represents a substantial advancement in RNA Nm site prediction. Future research directions include further optimization of the model architecture, such as exploring other machine learning approaches like contrastive learning [66] or diffusion models [67]. We also aim to integrate more RNA structural information to enhance feature representation. As new experimental data becomes available, we plan to update our dataset to further improve model performance. To address the interpretability issue, we intend to explore advanced explainable AI techniques and post-hoc explanation methods like SHAP [68], which could provide valuable biological insights into Nm modification mechanisms. Furthermore, we envision extending Nmix’s applications to other types of RNA modifications and related fields, such as RNA subcellular localization prediction [69] and RNA-protein interaction prediction [70]. These advancements may enhance our understanding of RNA biology and contribute to the study of related diseases. With further refinement and broader application, Nmix has the potential to play a crucial role in advancing our understanding of RNA modifications and their impact on various biological processes.

**Key Points**

- We introduced Nmix, a novel hybrid deep learning model for precise prediction of Nm sites in human RNA, significantly outperforming existing SOTA methods across various metrics, particularly in PRE and MCC.
- Nmix utilizes a newly constructed, large-scale, low-redundancy dataset of experimentally verified Nm sites, integrating data from multiple sources including RMBase v3.0 and recent experimental studies.
- The model employs an innovative multi-feature fusion approach combining one-hot encoding, Z-curve theory and RSS information, along with a sophisticated hybrid neural network architecture integrating 1D/2D CNNs, multi-head self-attention mechanism and residual connections.
- Nmix demonstrates excellent cross-species generalization capability, accurately predicting 93.8% of experimentally verified Nm sites in rat RNA.
- Nmix is now available as a web server at https://tubic.org/Nm, providing researchers with a valuable tool for RNA Nm site prediction.

## Supplementary Material

Supplementary_File_bbae601

## Data Availability

The benchmark dataset proposed in our study, the standalone prediction code, and detailed guides for their usage are publicly accessible at https://github.com/tubic/Nmix.
