# Supplementary material for: Nmix: a hybrid deep learning model for precise prediction of 2’-O-methylation sites based on multi-feature fusion and ensemble learning
Source: Brief Bioinform. 2024 Nov 16;25(6):bbae601. doi: 10.1093/bib/bbae601 (PMC11568878; doi:10.1093/bib/bbae601)
Supplement: Supplementary_File_bbae601 [file supplementary_file_bbae601.pdf]

Table S1. Sample counts in the benchmark dataset.

Subset	Type	Positive	Negative									
Am	Training set	1148	TS_1	TS_2	TS_3	TS_4	TS_5	TS_6	TS_7	TS_8	TS_9	TS_10
			1148	2296	3444	4592	5740	6888	8036	9184	10332	11480
	Validation set	181	1810									
	Independent test set	592	5920									
Cm	Training set	1098	TS_1	TS_2	TS_3	TS_4	TS_5	TS_6	TS_7	TS_8	TS_9	TS_10
			1098	2196	3294	4392	5490	6588	7686	8784	9882	10980
	Validation set	183	1830									
	Independent test set	538	5380									
Cm	Training set	1429	TS_1	TS_2	TS_3	TS_4	TS_5	TS_6	TS_7	TS_8	TS_9	TS_10
			1429	2858	4287	5716	7145	8574	10003	11432	12861	14290
	Validation set	233	2330									
	Validation set	667	6670									
Um	Training set	1146	TS_1	TS_2	TS_3	TS_4	TS_5	TS_6	TS_7	TS_8	TS_9	TS_10
			1146	2292	3438	4584	5730	6876	8022	9168	10314	11460
	Validation set	207	2070									
	Independent test set	615	6150									
Total	Training set	4821	TS_1	TS_2	TS_3	TS_4	TS_5	TS_6	TS_7	TS_8	TS_9	TS_10
			4821	9642	14463	19284	24105	28926	33747	38568	43389	48210
	Validation set	804	8040									
	Independent test set	2412	24120									

Table S2. Differences in chemical structures of four nucleotides.

Chemical structure	Attribute	Nucleotides
Ring Structure	Purine	A, G
	Pyrimidine	C, U
Functional Group	Amino	A, C
	Keto	G, U
Hydrogen Bond	Weak	A, U
	Strong	G, C

Table S3. Results of feature ablation study for Nmix-Specific.

Nm Type	Dataset	Model	ACC (%)	Recall (%)	PRE (%)	AUROC (%)	MCC (%)	F1-score (%)
Am	Validation set	One-hot	80.4	82.8	35.4	88.7	45.3	47.9
		Z-curve	62.9	87.7	18.6	81.5	28.2	30.6
		RSS	9.4	99.8	9.1	51.4	0.3	16.7
		One-hot + Z-curve	80.1	86.0	48.3	88.1	53.8	56.3
		One-hot + RSS	84.6	89.9	43.9	94.0	55.8	57.2
		Z-curve + RSS	60.8	85.6	18.1	77.1	26.2	29.7
		One-hot + Z-curve + RSS (Nmix)	**98.8**	**97.0**	**90.9**	**99.8**	**93.3**	**93.9**
	Independent test set	One-hot	81.1	83.1	36.8	89.0	46.7	49.2
		Z-curve	63.3	86.3	18.6	81.3	27.8	30.5
		RSS	9.3	100.0	9.1	51.8	0.6	16.7
		One-hot + Z-curve	80.1	83.5	48.8	87.4	53.0	56.0
		One-hot + RSS	85.4	90.7	46.4	94.8	58.0	59.3
		Z-curve + RSS	61.4	84.0	18.3	77.1	25.9	29.7
		One-hot + Z-curve + RSS (Nmix)	**99.3**	**97.0**	**95.1**	**99.9**	**95.7**	**96.1**
Cm	Validation set	One-hot	93.9	62.5	68.1	91.7	61.7	64.6
		Z-curve	92.4	48.0	58.7	89.3	48.9	52.4
		RSS	90.9	0.0	0.0	55.4	0.0	0.0
		One-hot + Z-curve	95.0	73.7	72.1	94.5	70.0	72.7
		One-hot + RSS	91.7	49.7	55.4	86.8	47.9	52.2
		Z-curve + RSS	90.7	28.5	41.8	84.1	29.7	33.3

Table S3. Results of feature ablation study for Nmix-Specific (continued 1).

Nm Type	Dataset	Model	ACC (%)	Recall (%)	PRE (%)	AUROC (%)	MCC (%)	F1-score (%)
Cm	Validation set	One-hot + Z-curve + RSS (Nmix)	**98.3**	**92.6**	**89.3**	**99.4**	**89.9**	**90.7**
	Independent test set	One-hot	94.1	64.7	69.4	92.0	63.6	66.6
		Z-curve	93.1	54.6	60.8	91.0	53.8	57.2
		RSS	90.9	0.0	0.0	53.6	0.0	0.0
		One-hot + Z-curve	95.3	74.1	73.6	94.6	71.2	73.7
		One-hot + RSS	92.3	53.6	58.5	88.4	51.7	55.7
		Z-curve + RSS	90.7	33.6	41.9	87.1	32.5	36.8
		One-hot + Z-curve + RSS (Nmix)	**98.5**	**94.5**	**89.5**	**99.4**	**91.1**	**91.9**
Gm	Validation set	One-hot	84.7	75.2	37.8	88.0	45.8	49.3
		Z-curve	66.7	68.8	17.0	73.4	21.0	27.3
		RSS	66.3	60.9	20.3	74.3	20.5	27.5
		One-hot + Z-curve	91.2	84.6	54.8	93.9	63.4	65.7
		One-hot + RSS	83.5	77.0	37.4	87.6	45.7	49.2
		Z-curve + RSS	70.4	64.0	20.8	73.7	23.4	30.3
		One-hot + Z-curve + RSS (Nmix)	**94.4**	**89.4**	**64.5**	**97.0**	**73.1**	**74.8**
	Independent test set	One-hot	83.4	72.5	35.8	86.0	42.8	46.9
		Z-curve	66.2	66.1	16.4	72.3	19.3	26.2
		RSS	60.4	65.0	16.6	73.9	17.1	24.9
		One-hot + Z-curve	91.2	83.3	55.5	93.1	63.3	65.8

Table S3. Results of feature ablation study for Nmix-Specific (continued 2).

Nm Type	Dataset	Model	ACC (%)	Recall (%)	PRE (%)	AUROC (%)	MCC (%)	F1-score (%)
Gm	Independent test set	One-hot + RSS	83.0	75.5	36.8	86.6	44.4	48.1
		Z-curve + RSS	70.3	59.3	20.0	71.7	20.9	28.8
		One-hot + Z-curve + RSS (Nmix)	**95.0**	**88.5**	**67.5**	**97.4**	**74.6**	**76.4**
Um	Validation set	One-hot	90.0	64.2	47.7	89.4	49.7	53.9
		Z-curve	91.5	36.5	33.0	82.7	32.0	34.6
		RSS	90.9	0.0	0.0	39.3	0.0	0.0
		One-hot + Z-curve	92.3	64.3	59.3	90.5	57.1	60.6
		One-hot + RSS	87.9	70.9	41.3	89.7	48.0	52.0
		Z-curve + RSS	90.4	16.0	13.6	72.8	12.5	14.7
		One-hot + Z-curve + RSS (Nmix)	**93.3**	**80.9**	**60.0**	**95.6**	**66.1**	**68.9**
	Independent test set	One-hot	89.5	62.7	46.0	89.6	47.8	52.3
		Z-curve	92.0	38.2	35.6	82.9	34.4	36.7
		RSS	90.9	0.0	0.0	45.7	0.0	0.0
		One-hot + Z-curve	92.4	66.2	60.3	91.0	58.6	62.0
		One-hot + RSS	88.3	71.9	42.7	90.4	49.4	53.2
		Z-curve + RSS	90.7	16.3	15.5	73.7	13.8	15.8
		One-hot + Z-curve + RSS (Nmix)	**93.5**	**83.4**	**60.5**	**95.8**	**67.7**	**70.1**

Note: The highest score in each column is shown in bold.

Table S4. Results of model architecture ablation study for Nmix-Specific.

Nm Type	Dataset	Model	ACC (%)	Recall (%)	PRE (%)	AUROC (%)	MCC (%)	F1-score (%)
Am	Validation set	Nmix-NoCNN	50.7	81.8	13.6	72.2	17.2	23.2
		Nmix-SingleCNN	75.9	84.4	25.5	88.2	37.3	39.1
		Nmix-NoAttention	72.8	78.5	22.5	83.6	31.5	34.9
		Nmix-NoResidual	74.4	81.7	29.9	84.1	38.4	41.9
		Nmix-MLP3	77.5	82.2	36.9	85.7	44.6	47.9
		Nmix-MLP5	79.0	89.3	49.5	89.6	56.2	58.9
		Nmix-NoFocal	94.2	91.0	67.6	97.5	75.1	76.4
		Nmix-Full	**98.8**	**97.0**	**90.9**	**99.8**	**93.3**	**93.9**
	Independent test set	Nmix-NoCNN	50.5	82.5	13.6	71.2	17.5	23.3
		Nmix-SingleCNN	76.2	82.9	25.6	87.9	37.0	39.0
		Nmix-NoAttention	73.3	77.0	22.5	83.1	31.2	34.7
		Nmix-NoResidual	74.7	79.5	29.9	83.5	37.7	41.7
		Nmix-MLP3	77.6	81.7	37.2	86.1	44.6	47.9
		Nmix-MLP5	79.6	88.7	52.1	89.3	57.6	60.5
		Nmix-NoFocal	95.0	92.1	72.0	97.7	78.4	79.6
		Nmix-Full	**99.3**	**97.0**	**95.1**	**99.9**	**95.7**	**96.1**
Cm	Validation set	Nmix-NoCNN	90.6	15.2	43.2	75.8	21.3	21.9
		Nmix-SingleCNN	90.9	52.1	51.4	87.2	46.6	51.3
		Nmix-NoAttention	90.6	50.2	49.4	85.4	44.4	49.3
		Nmix-NoResidual	91.6	49.4	54.8	87.6	47.3	51.6
		Nmix-MLP3	93.1	58.0	63.6	87.4	56.8	60.2
		Nmix-MLP5	95.2	70.8	73.1	93.8	69.2	71.5

Table S4. Results of model architecture ablation study for Nmix-Specific (continued 1).

Nm Type	Dataset	Model	ACC (%)	Recall (%)	PRE (%)	AUROC (%)	MCC (%)	F1-score (%)
Cm	Validation set	Nmix-NoFocal	92.5	62.3	58.3	90.9	56.1	60.1
		Nmix-Full	**98.3**	**92.6**	**89.3**	**99.4**	**89.9**	**90.7**
	Independent test set	Nmix-NoCNN	90.5	14.7	42.3	79.0	20.7	21.3
		Nmix-SingleCNN	91.0	52.2	51.2	87.4	46.6	51.2
		Nmix-NoAttention	91.3	54.1	53.2	86.0	48.7	53.2
		Nmix-NoResidual	92.1	51.8	57.5	88.6	50.1	54.1
		Nmix-MLP3	93.5	60.6	65.0	87.7	59.1	62.3
		Nmix-MLP5	95.5	72.9	74.5	94.1	71.1	73.2
		Nmix-NoFocal	92.9	63.6	60.9	90.9	58.3	62.1
		Nmix-Full	**98.5**	**94.5**	**89.5**	**99.4**	**91.1**	**91.9**
Gm	Validation set	Nmix-NoCNN	66.3	52.8	14.4	64.8	12.7	22.3
		Nmix-SingleCNN	84.4	72.9	35.7	87.4	43.5	47.3
		Nmix-NoAttention	78.7	67.0	25.4	81.2	31.6	36.6
		Nmix-NoResidual	84.4	81.1	38.7	89.7	48.8	51.5
		Nmix-MLP3	80.1	67.6	27.7	80.9	34.1	39.0
		Nmix-MLP5	89.8	80.4	50.9	91.2	58.6	61.5
		Nmix-NoFocal	**94.6**	**90.4**	**66.7**	**97.6**	**74.8**	**76.3**
		Nmix-Full	94.4	89.4	64.5	97.0	73.1	74.8
	Independent test set	Nmix-NoCNN	66.3	50.4	13.9	64.6	11.4	21.3
		Nmix-SingleCNN	82.0	66.0	30.8	82.7	36.3	41.4
		Nmix-NoAttention	77.6	64.8	23.9	79.2	29.2	34.7
		Nmix-NoResidual	83.5	75.8	36.6	87.5	44.8	48.5
		Nmix-MLP3	79.0	65.1	25.8	79.6	31.3	36.8

Table S4. Results of model architecture ablation study for Nmix-Specific (continued 2).

Nm Type	Dataset	Model	ACC (%)	Recall (%)	PRE (%)	AUROC (%)	MCC (%)	F1-score (%)
Gm	Independent test set	Nmix-MLP5	89.3	78.8	50.5	91.2	57.3	60.5
		Nmix-NoFocal	94.8	88.1	**68.3**	97.2	**74.7**	**76.5**
		Nmix-Full	**95.0**	**88.5**	67.5	**97.4**	74.6	76.4
Um	Validation set	Nmix-NoCNN	89.1	8.4	13.5	69.9	6.9	10.2
		Nmix-SingleCNN	86.1	55.0	36.5	84.9	37.1	42.9
		Nmix-NoAttention	88.3	56.2	40.0	84.8	41.1	46.6
		Nmix-NoResidual	90.0	58.3	48.5	88.7	47.0	51.1
		Nmix-MLP3	87.6	49.6	38.1	83.5	36.6	42.7
		Nmix-MLP5	91.1	67.0	52.2	90.1	54.2	58.2
		Nmix-NoFocal	**93.9**	76.1	**64.3**	95.4	**66.6**	**69.5**
		Nmix-Full	93.3	**80.9**	60.0	**95.6**	66.1	68.9
	Independent test set	Nmix-NoCNN	89.5	8.8	16.0	70.9	8.3	11.2
		Nmix-SingleCNN	87.6	58.9	40.5	86.5	41.9	47.1
		Nmix-NoAttention	88.0	52.6	38.4	84.0	38.4	44.2
		Nmix-NoResidual	90.1	59.7	50.0	88.9	48.5	52.5
		Nmix-MLP3	87.6	50.0	38.4	82.5	36.9	43.1
		Nmix-MLP5	91.0	66.5	52.1	89.6	53.8	57.8
		Nmix-NoFocal	93.7	76.2	**63.7**	95.3	66.1	69.0
		Nmix-Full	**93.5**	**83.4**	60.5	**95.8**	**67.7**	**70.1**

Note: The highest score in each column is shown in bold.

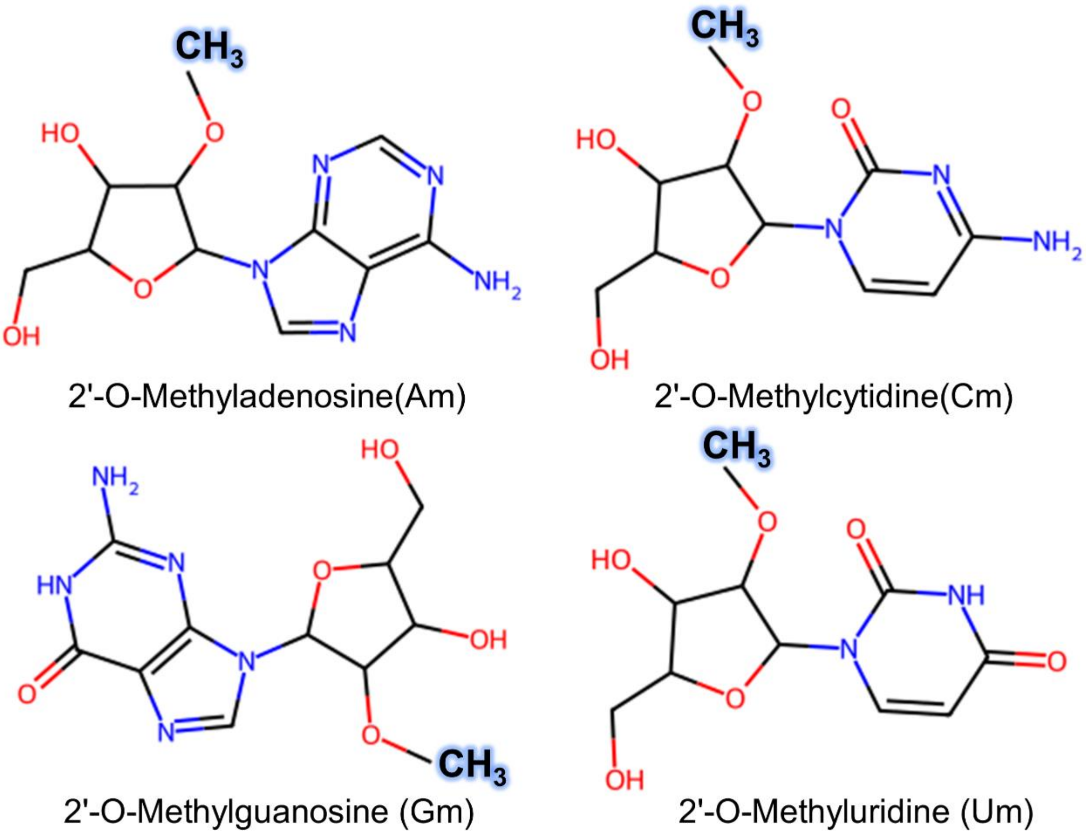

Fig. S1. Chemical structures of four 2'-O-methylated canonical nucleosides.

Supplementary information: Detailed architecture of CNN blocks

Our model incorporates both 1D and 2D CNN blocks to process different encodings of RNA sequences effectively. For the one-hot encoding (41×4) and Z-curve encoding (41×3), we implement two-layer 1D CNN blocks. The first layer employs 32 convolution kernels of size 3, transforming the input from 41×4 (or 41×3) to 41×32. The second layer uses 64 convolution kernels of size 5, further processing the features to a 41×64 dimension. This architecture allows the model to capture both local and more expansive patterns in the sequence data, with the increasing number of kernels enabling the network to learn a broader set of features as it progresses through the layers.

For the RSS encoding (41×41×1), we employ a two-layer 2D CNN block. Both layers use 3×3 convolution kernels, with the first layer containing 8 kernels and the second layer 16 kernels, transforming the input sequentially from 41×41×1 to 41×41×8, and then to 41×41×16. This design is particularly well-suited for extracting hierarchical spatial features from the 2D representation of RNA secondary structures, maintaining spatial relationships in the data while increasing the depth of features.

In both 1D and 2D CNN blocks, each convolutional layer is followed by batch normalization to stabilize the learning process and accelerate training. Batch normalization helps mitigate the internal covariate shift problem, potentially leading to faster convergence and improved generalization. The progressive increase in the number of filters allows the network to learn an increasingly rich set of features as it moves through the layers, enhancing its capability to capture complex patterns in the RNA sequence encodings.

We utilize the Parametric Rectified Linear Unit (PReLU) activation function after each convolution and batch normalization step. Unlike traditional ReLU, PReLU introduces a learnable

parameter that adapts during training, allowing for negative values with a small slope. This adaptability enables the model to learn more nuanced and complex representations of the data. The combination of carefully selected kernel sizes, increasing filter numbers, batch normalization, and adaptive activation functions enhances our model's ability to effectively capture and process diverse features from the multifaceted RNA sequence encodings, contributing to its overall performance in Nm site prediction tasks.

Supplementary information: Detailed explanations of performance evaluation metrics

$$\begin{cases} ACC = \dfrac{TP + TN}{TP + TN + FP + FN} \\ Recall = \dfrac{TP}{TP + FN} \\ PRE = \dfrac{TP}{TP + FP} \\ MCC = \dfrac{TP \times TN - FP \times FN}{\sqrt{(TP + FP)(TP + FN)(TN + FP)(TN + FN)}} \\ AUROC = \int TPRd(FPR) \\ F1 - score = 2 \times \dfrac{PRE \times Recall}{PRE + Recall} \end{cases}$$

In the realm of machine learning, particularly in classification tasks, a diverse array of metrics is employed to comprehensively evaluate model performance. These metrics are primarily derived from four fundamental components of the confusion matrix: True Positive (TP), True Negative (TN), False Positive (FP), and False Negative (FN). TP represents instances where the model correctly identifies positive cases, while TN denotes accurate classification of negative instances. Conversely, FP occurs when the model erroneously classifies negative instances as positive, and FN represents positive instances misclassified as negative.

Accuracy (ACC) measures the overall precision of the model across all classes. However, in scenarios with imbalanced datasets, ACC alone may not provide a complete picture of model performance. This limitation necessitates the use of more nuanced metrics. Recall, also known as Sensitivity (SEN) or True Positive Rate (TPR). It quantifies the model's proficiency in identifying positive instances, making it particularly valuable in applications where missing positive cases could have serious consequences. Precision (PRE) evaluates the accuracy of positive predictions. It is especially significant in contexts where false positives carry substantial risks or costs. The Matthews Correlation Coefficient (MCC) provides a balanced measure of the quality of binary classifications.

Its values range from -1 to +1, where +1 indicates perfect prediction, 0 suggests no better than random prediction, and -1 implies total disagreement between prediction and observation. MCC is particularly useful when dealing with imbalanced datasets as it considers all four confusion matrix categories. The Area Under the Receiver Operating Characteristic curve (AUROC) assesses the model's discriminative capability across various threshold settings. It plots the True Positive Rate against the False Positive Rate (FPR = FP / (FP + TN)) at different classification thresholds. An AUROC of 1.0 represents perfect classification, while 0.5 indicates performance no better than random guessing. The F1-score represents the harmonic mean of PRE and Recall. This metric is particularly useful for imbalanced datasets as it provides a balanced measure of the model's performance in terms of both its precision in positive predictions and its sensitivity in detecting positive instances.
